# Variation in Yield Gap Induced by Nitrogen, Phosphorus and Potassium Fertilizer in North China Plain

**DOI:** 10.1371/journal.pone.0082147

**Published:** 2013-12-12

**Authors:** Xiaoqin Dai, Zhu Ouyang, Yunsheng Li, Huimin Wang

**Affiliations:** Key Laboratory of Ecosystem Network Observation and Modeling, Institute of Geographic Sciences and Natural Resources Research, Chinese Academy of Sciences, Beijing, China; University of Illinois, United States of America

## Abstract

A field experiment was conducted under a wheat-maize rotation system from 1990 to 2006 in North China Plain (NCP) to determine the effects of N, P and K on yield and yield gap. There were five treatments: NPK, PK, NK, NP and a control. Average wheat and maize yields were the highest in the NPK treatment, followed by those in the NP plots among all treatments. For wheat and maize yield, a significant increasing trend over time was found in the NPK-treated plots and a decreasing trend in the NK-treated plots. In the absence of N or P, wheat and maize yields were significantly lower than those in the NPK treatment. For both crops, the increasing rate of the yield gap was the highest in the P omission plots, i.e., 189.1 kg ha^−1^ yr^−1^ for wheat and 560.6 kg ha^−1^ yr^−1^ for maize. The cumulative omission of P fertilizer induced a deficit in the soil available N and extractable P concentrations for maize. The P fertilizer was more pivotal in long-term wheat and maize growth and soil fertility conservation in NCP, although the N fertilizer input was important for both crops growth. The crop response to K fertilizers was much lower than that to N or P fertilizers, but for maize, the cumulative omission of K fertilizer decreased the yield by 26% and increased the yield gap at a rate of 322.7 kg ha^−1^ yr^−1^. The soil indigenous K supply was not sufficiently high to meet maize K requirement over a long period. The proper application of K fertilizers is necessary for maize production in the region. Thus, the appropriate application of N and P fertilizers for the growth of both crops, while regularly combining K fertilizers for maize growth, is absolutely necessary for sustainable crop production in the NCP.

## Introduction

The North China Plain (NCP) is the largest and the most important agricultural production region in China. It covers approximately 18.3% of total national farm lands (18 million hectares) and produces 21.6% of the total grain yield of edible crops in the country [1]. The main crops grown are wheat and maize, periodically rotated. The NCP now supplies more than 50% of the nation’s wheat and 33% of its maize [2]. Grain production and the maintenance of soil fertility in the NCP are very important for the food security and agricultural sustainability of China.

Nutrient availability is the most yield-limiting factor. To produce higher yields, the over-application of chemical fertilizers has been a common practice in wheat-maize rotation systems and has led to severe environmental problems [3]. Improved nutrient management practices are urgently needed to maximize crop yields and maintain soil fertility while minimizing environmental impacts. The ability to better identify crop response to the application of fertilizers, soil indigenous nutrient supply capability, and the maintenance of soil fertility over time are crucial to the development of improved nutrient management practices. Various long-term experiments have been conducted to test the effects of fertilization on yield or soil fertility throughout the world [4]–[14], but over long time scales, crop response to the application of N, P and K fertilizers and soil indigenous nutrient supply capability has seldom been clearly understood.

The yield and soil fertility gap between a full NPK fertilizer plot and a fertilizer omission plot was used as a good diagnostic tool to assess the extent of macronutrient limitations. Mussgnug et al (2006) analyzed the yield gap resulting from nutrient limitation on a degraded soil in the Red River Delta (RRD) in Northern Vietnam. They found that in the absence of K application, the yield gap for rice and maize respectively averaged 1.7 Mg ha^−1^ and 3.4 Mg ha^−1^, while when N or P was omitted the yield gap was less. Potassium was the most yield-limiting macronutrient in RRD of Vietnam [15]. The factors that primarily limit increasing crop yields varied by crop and region [16]. The objectives of the study were to examine the effects of the continuous use of inorganic fertilizers on wheat and maize yield and soil fertility gap to elucidate crop response to fertilizer inputs and the long-term N, P and K supply capability in the NCP.

## Materials and Methods

### Experimental Site

A long-term field experiment was conducted from 1990 to 2006 at the Yucheng Comprehensive Experimental Station (36°57′ N, 116°36′ E, 28 m a.m.s.l.) of the Chinese Academy of Sciences (CAS) located in Shandong Province, which lies in the NCP. This area represents the moderate- to high-yielding region of the NCP, where the dominant cropping system is double-cropped wheat-maize. The annual mean precipitation is 575 mm, with approximately 70% of the total precipitation falling between June and September. The soil is classified as fluvo-aquic loam soil. Before the initiation of the experiment, soil samples were collected from depths of 0–20 cm and 20–40 cm by stepwise soil augers in the experimental field in 1990. All samples from the same soil layers were mixed thoroughly to create one homogenous sample, and a representative sample was drawn to determine the soil texture, bulk density, organic carbon, total N, P and K, and available N, P and K. The physical and chemical properties of the soil in 1990 are listed in Table 1.

**Table 1:** Physical and chemical properties of soil at different layers at Yucheng, Shandong Province of China in 1990.

Soil parameters	0–20 cm	20–40 cm
Soil texture	Silt loam	Sandy loam
Clay (%)	17.3	13.7
Silt (%)	61.4	11.4
Sand (%)	21.3	74.9
Bulk density (g cm^−3^)	1.31	1.48
Field capacity (%)	23.3	22.7
Organic C (g kg^−1^)	5.4	4.8
Total N (g kg^−1^)	0.41	0.36
Total P (g kg^−1^)	1.11	1.13
Total K (g kg^−1^)	10.7	14.0
Available N (mg kg^−1^)	46.1	41.4
Available P (mg kg^−1^)	8.3	5.4
Available K (mg kg^−1^)	149.0	125.4

### Experimental Design and Treatments

The study consisted of five treatments: no fertilization (control); N, P, and K applied (NPK); N and P applied (NP; K omission); N and K applied (NK; P omission); P and K applied (PK; N omission), with four replications performed for each condition in a randomized complete block design (Table 2). The application rates of N, P and K, if they were applied, were the same in all of the treatments: 253 kg N ha^−1^ as urea (46% N), 90 kg P_2_O_5_ ha^−1^ as single superphosphate (16% P_2_O_5_), and 171 kg K_2_O ha^−1^ as potassium sulfate (50% K_2_O) for winter wheat and 262 kg N ha^−1^ as urea (46% N), 52 kg P_2_O_5_ ha^−1^ as single superphosphate (16% P_2_O_5_), and 298 kg K_2_O ha^−1^ as potassium sulfate (50% K_2_O) for maize (Table 2). Phosphate was applied before sowing and incorporated during land preparation. Winter wheat received 40% of the total N as a basal dressing before sowing, whereas the remainder was top-dressed in two splits (40% at green-up and 20% at flowering). Approximately 50% of the K was applied before sowing and 50% top-dressed at green-up (the first leaf growing in the early of spring growed to 1–2 cm from soil surface and the phenomena was seen on a half of wheat seedling in the field). During the maize season, all of the P and K fertilizers and 35% of the N fertilizer were applied as a basal fertilizer, and 65% of the N was applied at the elongation stage. Each plot area measured 6 m×5 m, and all plots were isolated from one another by a concrete wall to a soil depth of 1 m.

**Table 2:** Treatment and fertilizer nutrient rates (kg ha^−1^) applied to winter wheat and maize at Yucheng, Shandong Province of China.

Treatments	Winter wheat			Maize		
	N	P_2_O_5_	K_2_O	N	P_2_O_5_	K_2_O
NPK	253	90	171	262	52	298
PK	0	90	171	0	52	298
NK	253	0	171	262	0	298
NP	253	90	0	262	52	0
Control	0	0	0	0	0	0

Winter wheat (*Triticum aestivum*) was double-cropped with maize (*Zea mays*), and the crops were grown along the border of the four blocks. Winter wheat was sown in mid-October and harvested in early June of the next year. Maize was sown in mid-June and harvested in early October of the same year. The winter wheat was seeded at a rate of 225 kg ha^−1^, and the maize was planted at a rate of 75000 seeds ha^−1^. Surface irrigations were conventionally conducted according to the conditions of crop growth and soil moisture. Wheat and maize were harvested to the level of the soil surface; thus, the stubble left in the field was negligible. However, the roots were left in the soil. All straws were removed from the field. All plots were kept free of weeds by the application of an herbicide. Pesticides were applied in accordance with good practices for crop protection.

### Sampling and Chemical Analyses

At harvestable maturity, the grain yields were determined over the whole plot area. Soils samples were collected at a depth of 20 cm at six randomly selected points in every plot after wheat and maize were harvested each year. The soil samples were mixed thoroughly from all cores to obtain a representative soil sample for each plot. The soil samples were air-dried and sieved through a 1-mm mesh to perform available N, Olsen-P and available K measurements and through a 0.149-mm mesh to estimate the organic carbon and total N content. Soil available N was determined using the alkali-hydrolytic diffusion method [17]. Olsen-P was measured by ascorbic acid-molybdate blue colorimetry [18]. Available K was extracted with an ammonium acetate solution (NH_4_OAc, 1 mol/L) and then determined with a flame photometer [17]. Soil organic carbon was determined using the wet oxidation method of Walkley and Black, and total N was measured following Kjeldahl digestion and distillation [17].

### Data Analyses

The results of yield and soil fertility in the first three years of the experiment were not used because in the autumn of 1993 soybean was sown. Therefore, the results reported are from 1994 to 2006. Crop yield losses caused by N, P, K or NPK omission were calculated from the differences between an NPK treatment and the PK, NK, NP or control plot during the same years. The yield differences were called the “yield gap” induced by N (GYG_N_), P (GYG_P_), K (GYG_K_) or NPK (GYG_NPK_) fertilizer omission to assess the extent of macronutrient limitations in the study. The differences in soil available N, Olsen-P and available K between the NPK treatment and the PK, NK, NP or control plots were calculated to evaluate the variation in soil fertility when nutrients were limited. The soil nutrient differences were called the “soil nutrient gap (SNG)” induced by N (SNG_N_), P (SNG_P_), K (SNG_P_), or NPK (SNG_NPK_) fertilizer omission.

All statistical analyses including the analysis of variance and regression were conducted using the SPSS package (version 16.0). Considering the data over time was lack of independence, the repeated measures ANOVA between differences in mean through years among treatments for various parameters (grain yield, soil organic C, total N, available N, Olsen-P, available K) were analyzed using a Fisher’s protected least significant difference (LSD) test at *P* = 0.05. A mixed models with random block and block×treatment were done to assess trends (slopes) of grain yield, yield gap and various soil nutrients gap over the years. The *P*-values of the slopes were used to test whether the observed changes were significantly different from 0.

## Results

### Grain Yield and Yield Gap

**Figure 1:**
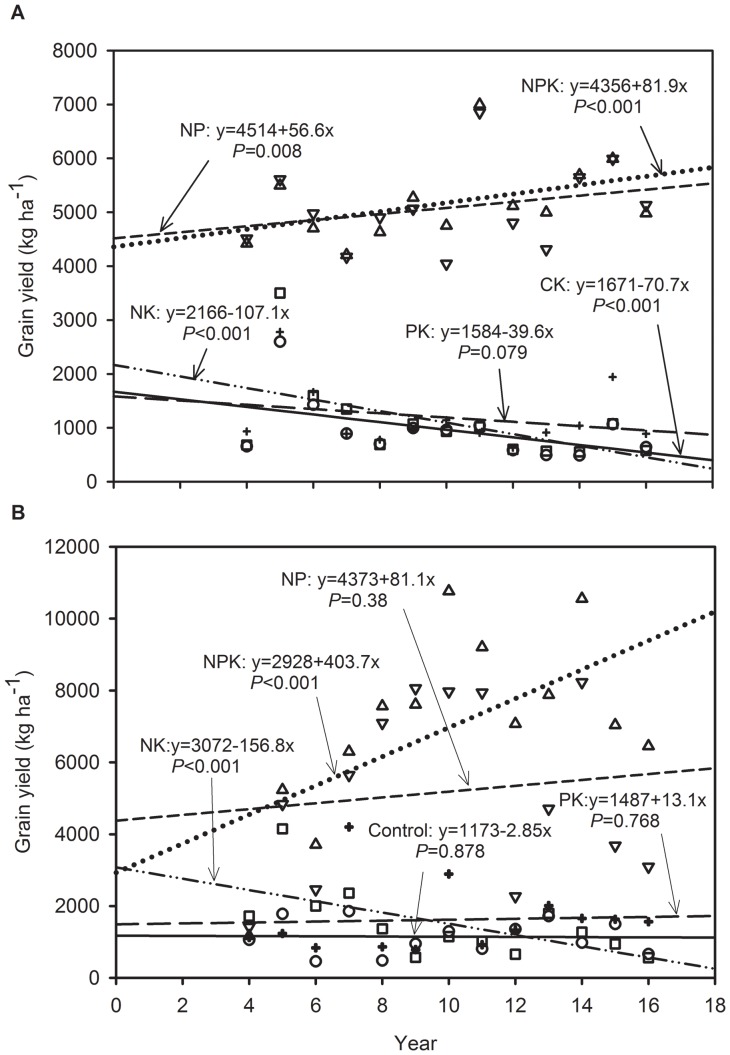
Grain yield of wheat (a) and maize (b) of different fertilizer treatments (Control, circle; NPK, triangle up; NP, triangle down; NK, square; PK, plus) under wheat-maize rotation system from 1994 to 2006 at Yucheng, Shandong Province of China.

The fertilizer treatments significantly affected the grain yields (Table 3). The wheat and maize yields were highest in the NPK treatment, followed by those in the NP treatment. In the PK or NK treatments, the yields of wheat and maize were significantly lower than those in the NP and NPK treatments. Compared to those in the NPK treatment, the yields of wheat and maize decreased, respectively, by 77% with the N omission, 79% and 78% with the P omission, 2% and 26% with the K omission, and 81% and 84% with no fertilizer. The grain yield losses were much greater in either N or P omission than in plots fertilized combined N and P. Over time wheat yield significantly increased at a rate of 81.9 kg ha^−1^ yr^−1^ in NPK plots (*P* = 0.008), and maize yield also significantly increased at a rate of 403.7 kg ha^−1 ^yr^−1^ in NPK plots (*P*<0.001, Fig. 1). The yields of wheat and maize in NK treatment decreased significantly at a rate of 107.1 kg ha^−1 ^yr^−1^ (*P*<0.001) and 156.8 kg ha^−1^ yr^−1^ (*P*<0.001) over time during the study period, respectively (Fig. 1). The long-term omission of K fertilizer also significantly reduced the maize yield compared to the maize yield of the NPK-treated plot (Table 3). But both crops yields had no significant upward or downward trends when K fertilizer was omitted (Fig. 1).

**Table 3:** Multi-year average grain yield of wheat and maize, as affected by various treatments with fertilizer in a wheat-maize rotation system at Yucheng, Shandong Province of China (mean±standard error).

Treatment	Wheat (kg ha^−1^)	Maize (kg ha^−1^)
Control	963±155 b	1145±133 c
NPK	5174±201 a	6965±724 a
NP	5080±216 a	5184±666 b
NK	1094±218 b	1504±270 c
PK	1188±165 b	1618±269 c

*P*<0.05 between fertilizer treatments. Different letters within a column indicate a significant difference at

**Figure 2:**
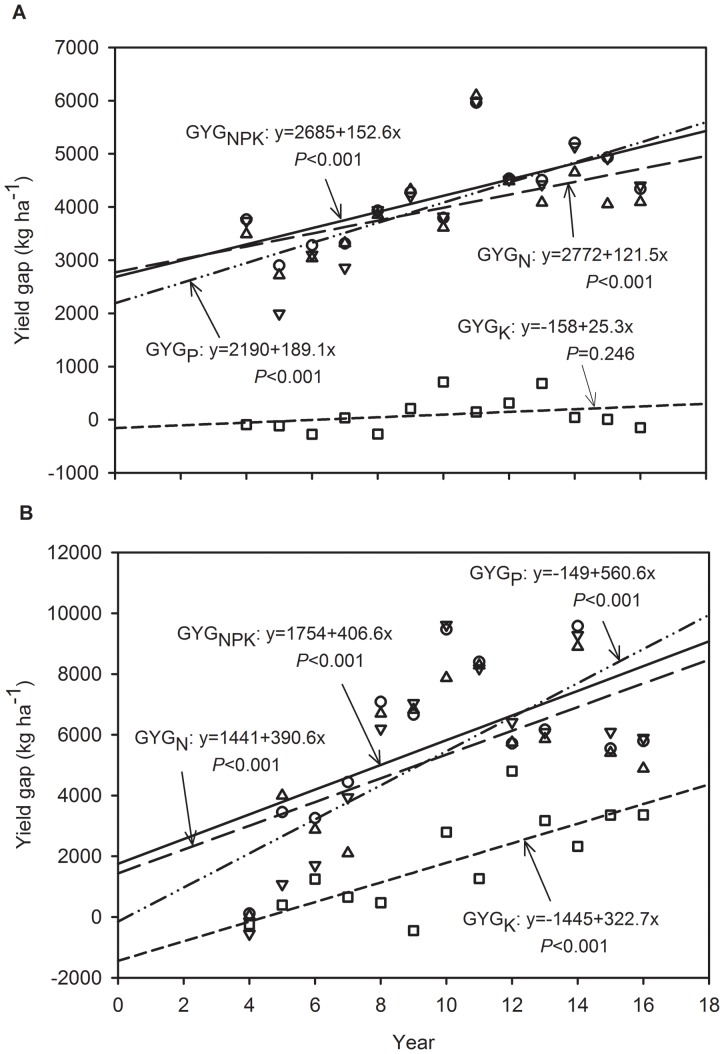
Yield gap variation of wheat (a) and maize (b) by N (GYG_N_, the yield difference between NPK and PK plots in each year, triangle up), P (GYG_P_, the difference between NPK and NK plots, triangle down), K (GYG_K_, the difference between NPK and NP plots, square) or NPK (GYG_NPK_, the difference between NPK and Control plots, circle) fertilizer omission from 1994 to 2006 at Yucheng, Shandong Province of China.

The variation in the yield gap of wheat and maize between the NPK and PK, NK, NP and control plots is illustrated in Figure 2. For wheat, the yield gap significantly increased at a rate of 152.6 kg ha^−1^ yr^−1^ for the control plots (*P*<0.001), a rate of 189.1 kg ha^−1^ yr^−1^ for the NK plots (*P*<0.001) and a rate of 121.5 kg ha^−1^ yr^−1^ for the PK plots (*P*<0.001). In the NP plots, the yield gap also gradually increased with the cumulative effect of K omission, but the relationship was not significant (*P* = 0.246). However, for maize, when the N, P K or NPK fertilizer was omitted, the yield gap significantly increased with the continuous omission of nutrients. The significant increase in the yield gap was 390.6 kg ha^−1^ yr^−1^ for the PK treatments (*P*<0.001), 560.6 kg ha^−1^ yr^−1^ for the NK treatments (*P*<0.001), 322.7 kg ha^−1^ yr^−1^ for the NP treatments (*P*<0.001), and 406.6 kg ha^−1^ yr^−1^ for the control treatments (*P*<0.001). For both crops, the increasing rate of the yield gap was highest in the P omission plots, which indicated that the cumulative effect of P omission was the most significant for wheat and maize in the region. The slopes of the yield gap were the second highest in the N omission plots for both crops, which indicated that N fertilizer inputs were important for wheat and maize growth, especially the latter. It is worth noting that the slopes of the maize yield gap increased significantly with continuous K omission. This result indicates that the soil indigenous K supply was not sufficiently high to meet the maize K requirement over a long period.

### Soil Nutrient Status

Long-term continuous treatment with different fertilizers significantly affected the soil organic carbon, available N, Olsen-P and available K concentrations (Table 4). The multi-year average soil organic carbon content was significantly lower in the control, NK and PK plots than in the NPK and NP plots for wheat and maize. The decrease was 6.7% to 15.6% for wheat and 7.4% to 15.8% for maize. The different treatments except control had no significant influences on soil total N. The available N, Olsen-P and available K concentrations were higher in the plots treated with the corresponding N, P or K fertilizer inputs (Table 4). For wheat, the available N concentration increased significantly in the acquired N fertilizer inputs plots compared to that in the PK plots. The Olsen-P concentration in plots with P fertilizer inputs increased significantly by 7.2 to 12.3 times the concentration in the control plots. Meanwhile, the Olsen-P concentration also increased by 9% and 62% in the NP and PK plots, respectively, compared with that in the NPK plots, indicating that the P uptake of the crops was lower in the NP and PK plots. Similar to the Olsen-P concentration, the available K concentration increased significantly in the plots with K fertilizer inputs, and the increase was as high as 1.2 to 2.3 times the K concentration in the control plots. In the NK and PK plots, the available K concentration increased by 48% and 55%, respectively, compared to that in the NPK plots.

**Table 4:** Multi-year average soil nutrient status at wheat and maize harvest, as affected by various treatments with fertilizer in a wheat-maize rotation system at Yucheng, Shandong Province of China (mean±standard error).

Crops	Treatments	Organic C (g kg^−1^)	Total N (g kg^−1^)	Available N (mg kg^−1^)	Olsen-P (mg kg^−1^)	Available K (mg kg^−1^)
Wheat	Control	5.5±0.19 c	0.56±0.04b	52.5±5.15b	3.1±0.28c	104.5±5.50c
	NPK	6.5±0.21a	0.66±0.05a	63.3±4.35a	25.2±1.95b	226.1±16.0b
	NP	6.3±0.15ab	0.67±0.05a	62.7±4.07a	27.5±2.04b	91.6±8.72c
	NK	5.9±0.17abc	0.64±0.05a	62.5±4.17a	3.2±0.31c	333.4±40.3a
	PK	5.8±0.19bc	0.56±0.04ab	49.1±4.20b	40.8±3.98a	349.3±35.3a
Maize	Control	5.7±0.13c	0.66±0.02ab	56.9±6.11b	2.7±0.32c	106.5±5.22c
	NPK	6.7±0.15a	0.71±0.05a	65.8±6.45a	22.1±2.47b	283.7±24.6b
	NP	6.5±0.15ab	0.69±0.04ab	66.6±6.05a	21.7±1.78b	90.9±4.46c
	NK	6.0±0.13bc	0.66±0.04ab	68.4±6.76a	3.6±0.54c	423.8±43.7a
	PK	5.8±0.14bc	0.62±0.03b	52.1±5.90b	33.5±2.32a	438.8±39.8a

*P*<0.05 between fertilizer treatments. For each crop, different letters within a column indicate a significant difference at

**Figure 3:**
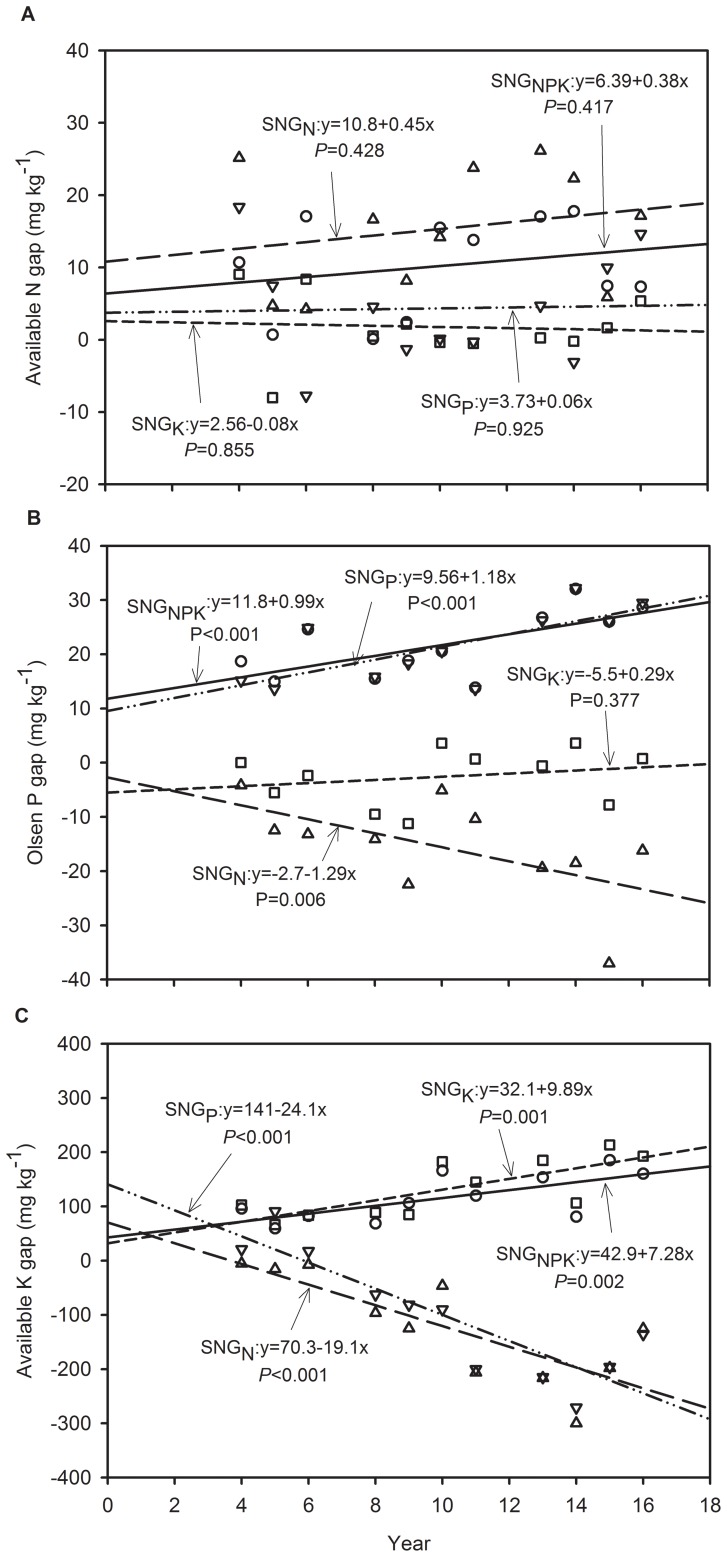
Soil available N (a), Olsen-P (b) and available K (c) gap variation by N (SNG_N_, soil available nutrient difference between NPK and PK plots, triangle up), P (SNG_P_, the difference between NPK and NK plots, triangle down), K (SNG_K_, the difference between NPK and NP plots, square) or NPK (SNG_NPK_, the difference between NPK and Control plots, circle) fertilizer omission from 1994 to 2006 during wheat season at Yucheng, Shandong Province of China.

**Figure 4:**
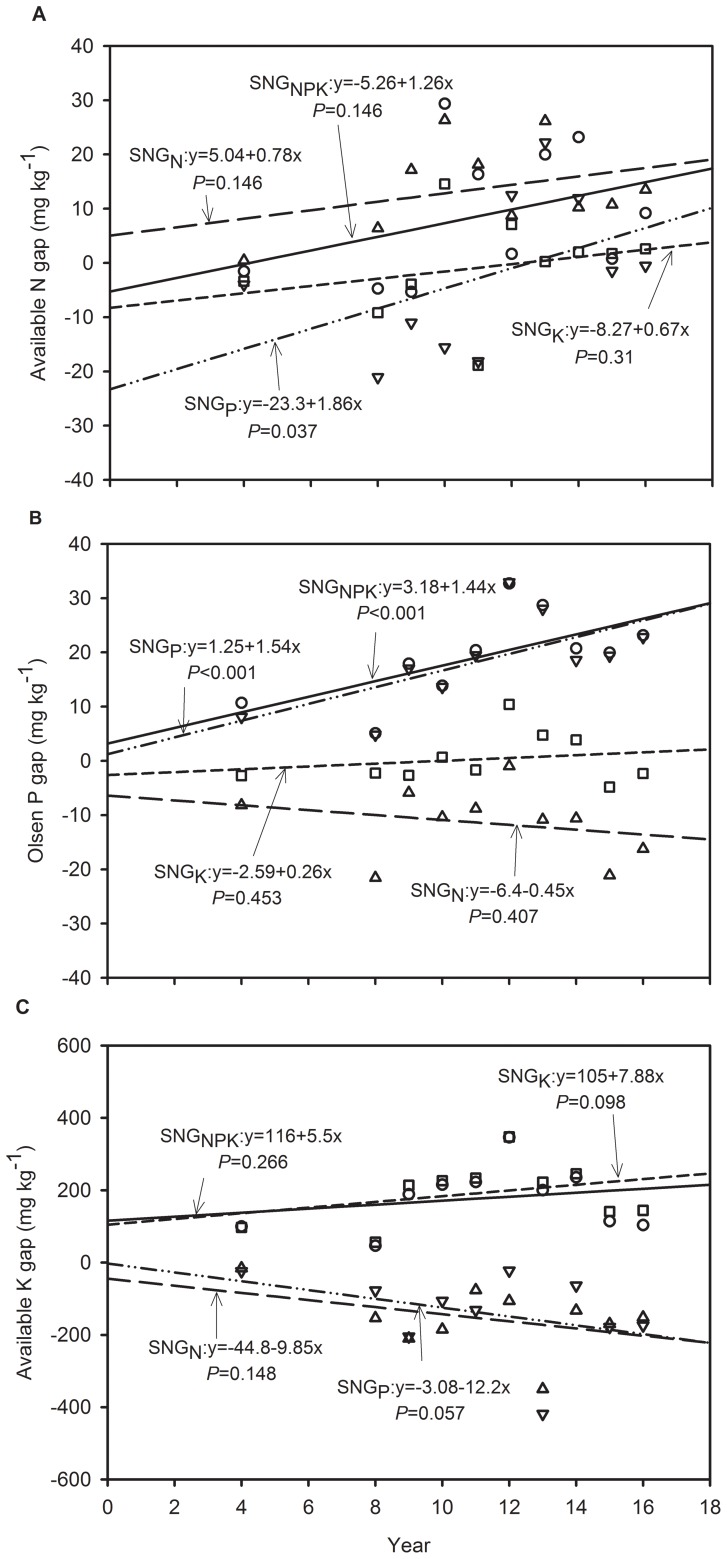
Soil available N (a), Olsen-P (b) and available K (c) gap variation by N (SNG_N_, soil available nutrient difference between NPK and PK plots, triangle up), P (SNG_P_, the difference between NPK and NK plots, triangle down), K (SNG_K_, the difference between NPK and NP plots, square) or NPK (SNG_NPK_, the difference between NPK and Control plots, circle) fertilizer omission from 1994 to 2006 during maize season at Yucheng, Shandong Province of China.

The differences in soil nutrients between the NPK and PK, NK, NP or NPK omission plots are shown in Figure 3&4. During the wheat growth season, the continuous omission of nutrients had no significant effects on the difference in the soil available N concentration (Fig. 3a). The continuous omission of P fertilizer significantly enhanced the differences in the soil Olsen-P concentration in the NPK plots (*P*<0.001), indicating that the long-term absence of P fertilizer resulted in a great deficit in the soil available P concentration. Similarly to the P omission plots, the control plots also possessed a significantly enhanced soil Olsen-P gap (*P*<0.001). However, a surplus concentration of Olsen-P was observed when the continuous N was omitted (*P* = 0.006, Fig. 3b). Also a surplus concentration of available K was observed when the continuous P and N inputs were omitted (*P*<0.001) due to a large amount of K fertilizer inputs and lower crop uptake. When the K fertilizer was omitted, the soil available K concentration gap significantly increased (*P* = 0.001 for NP and *P* = 0.002 for Control), indicating a deficit in soil available K in the NP and Control plots.

During the maize growth season, the soil available N concentration gradually became deficient when the nutrients were omitted, but only when P fertilizer was omitted, the effects were significant (Fig. 4a). Like wheat, P fertilizer omission induced a severe deficit in soil Olsen-P (*P*<0.001). Although the PK plots feature a larger amount of P fertilizer inputs and lower crop uptake, no significant surplus in the soil Olsen-P concentration was observed (Fig. 4b). In the plots fed K fertilizer inputs, a surplus in the soil available K concentration was observed, but the effects were not significant (Fig. 4c). It is worthy to note that the NP plots induced a deficit in soil available K (Table 4). Although the effects were not significant, maize growth was significantly affected due to the shortage of soil available K.

## Discussion

The response of wheat and maize growth of combined N and P fertilization (i.e. NP) was much greater than that of K fertilizer application combined with N or P (i.e. NK and PK treatments) (Table 3). The continuous 16-yr omission of N or P fertilizer significantly reduced the wheat and maize yield (Table 3). The grain yield in the N omission plots exhibited no significant decreasing trends over time (Fig. 1a). On the one hand, the continuous N omission significantly reduced the soil available N concentration, but no significant effects were observed on the total N concentration (Table 4). The differences in soil available N between the NPK and PK plots also exhibited no significant increase over time (Fig. 3a&4a). It might be possible that atmospheric nitrogen deposition in the NCP soil, N mineralization and lower nitrogen depletion may have attributed to the trend in available N over the years in the nitrogen omitted plots. Liu et al. (2006) and Zhang et al. (2008) reported that the bulk deposition of inorganic N in the NCP is approximately 30 kg/ha per year, which has a significant effect on agricultural systems [19], [20]. But yield gap significantly increased over time when the N was omitted (Fig. 2), indicating that the soil indigenous N supply combined with the deposition of N could not meet the N requirement for wheat and maize growth in the NCP.

Both of the wheat and maize yields had significant downward trends in P omission treatment (Fig. 1) and the yield gap of both crops significantly increased with the continuous omission of P fertilizer (Fig. 2), indicating that the cumulative absence of P fertilizers significantly inhibited wheat and maize growth. Tang et al. (2008) also reported that wheat and maize yields without P fertilization significantly decreased over time in several other sites in China [21]. The results are different from those reported under European conditions, most likely due to differences in the P supplying abilities of the different soils [22]. It is noted that the continuous omission of P fertilizer significantly reduced the soil Olsen-P concentration for both wheat and maize (Table 4). Meanwhile, the differences in the Olsen-P concentration between the NPK and NK plots significantly increased with continuous P omission (Fig. 3b&4b). The results indicate that the long-term absence of P fertilizer resulted in a great deficit in soil available P. It is interesting that the continuous omission of P fertilizer increased the available N concentration gap between the NPK and NK plots, and the effects were significant for maize (Fig. 4a), which indicates that a large amount of available N was depleted or lost, although the plots were fed N fertilizer inputs. The cumulative absence of P fertilizer intensified the shortage of N for crop growth. Because the treatments fertilized without P had a lower the number of cultivable microorganisms, microbial biomass and community functional diversity than in the treatments with P fertilization [23], which possibly induced the lower N and P mineralization in treatments fertilized without P.

The yield response to K fertilizer was much lower than that to N or P fertilizer (Table 3), most likely due to the high inherent soil K levels, which were in excess of the crop K demands. Most soils of the alluvial floodplain in Asia are high in K, and K is a rare limiting factor [24]. Shen et al. (2004) conducted a 14-yr field trial in Hebei Province of northern China and indicated that the application of N and P enhanced rice yields, while K had no yield-increasing effect due to the large resource of available soil K [25]. In our study, wheat and maize yield in the plots of combined NP and K fertilizer significantly improved, and the yield gap of maize significantly increased with continuous K omission; however, that of wheat yield did not (Fig. 1&2). Compared to that in the NPK plots, the soil available K concentration significantly decreased in the NP plots (Table 4). The differences in soil available K between NPK and NP also improved with the continuous omission of K, and the effects were significant during the wheat season (Fig. 3c). Although soil available K was gradually depleted, the soil indigenous K supply was sufficiently high to meet the requirement for normal wheat growth over the 16-yr period of the study. If the K fertilizer was continuously omitted for a longer period, the soil available K deficit would possibly inhibit wheat growth, which must be further verified. In addition, the soil available K gap between the NPK and NP plots during maize growth season showed an increasing trend with the omission of K, but the differences were not significant (Fig. 4c). This behavior is because the long-term omission of K fertilizer significantly reduced the maize yield, which resulted in lower K uptake. The results show that long-term maize production is much more sensitive to the absence of K than wheat production in the NCP. The results are supported by those of Tan et al. (2007), who concluded that the effect of K fertilizer on maize was higher than that on wheat under the wheat-maize rotation system of Hebei [26].

In our study, the combination of N and P fertilizers mostly sustained soil organic carbon, total N and available N, P and K levels over time (Table 4). This result is similar to that of Shen et al. (2004), who reported that the soil organic carbon and total N concentrations remained stable over time [25]. Manna et al. (2005) also showed that the recommended NPK plots are adequate for maintaining a constant SOC content under the sub-humid and semi-arid tropical conditions of India over a long period [27]. When N fertilizer was omitted, soil available P and K significantly improved in the plots with P and K fertilizer (PK). Meanwhile, when P fertilizer was omitted, soil available K was significantly accumulated in the plots with N and K fertilizer (NK, Table 4). Because the deficiency of other nutrients inhibited crop production, both crops exhibited lower P or K uptake. The application of inorganic fertilizer allowed the crops to exceed production needs and thus resulted in a substantial build-up of available P or available K, which inevitably induced resource waste and ecological pollution.

## Conclusions

The P fertilizer was more pivotal in the long-term growth of wheat and maize and the conservation of soil fertility in the NCP, although the N fertilizer input was important for the growth of both crops as well. Although the crop yield response to K fertilizer was much lower than to N or P fertilizer, the proper application of K fertilizer is also necessary, especially for maize production in the region. Thus, the appropriate application of N and P fertilizers for both crops, in combination with regular K fertilizers for maize, is absolutely necessary in terms of sustainable crop production in the NCP. However, a longer-range study is required to verify whether the soil indigenous K supply could continue to meet the requirement for wheat growth over many years.
